# Simple, sensitive, and cost-effective detection of *w*AlbB *Wolbachia* in *Aedes* mosquitoes, using loop mediated isothermal amplification combined with the electrochemical biosensing method

**DOI:** 10.1371/journal.pntd.0009600

**Published:** 2022-05-13

**Authors:** Parinda Thayanukul, Benchaporn Lertanantawong, Worachart Sirawaraporn, Surat Charasmongkolcharoen, Thanyarat Chaibun, Rattanalak Jittungdee, Pattamaporn Kittayapong

**Affiliations:** 1 Center of Excellence for Vectors and Vector-Borne Diseases, Faculty of Science, Mahidol University at Salaya, Nakhon Pathom, Thailand; 2 Department of Biology, Faculty of Science, Mahidol University, Bangkok, Thailand; 3 Biosensors Laboratory, Department of Biomedical Engineering, Faculty of Engineering, Mahidol University, Nakhon Pathom, Thailand; University of Florida, UNITED STATES

## Abstract

**Background:**

*Wolbachia* is an endosymbiont bacterium generally found in about 40% of insects, including mosquitoes, but it is absent in *Aedes aegypti* which is an important vector of several arboviral diseases. The evidence that *Wolbachia* trans-infected *Ae*. *aegypti* mosquitoes lost their vectorial competence and became less capable of transmitting arboviruses to human hosts highlights the potential of using *Wolbachia-*based approaches for prevention and control of arboviral diseases. Recently, release of *Wolbachia* trans-infected *Ae*. *aegypti* has been deployed widely in many countries for the control of mosquito-borne viral diseases. Field surveillance and monitoring of *Wolbachia* presence in released mosquitoes is important for the success of these control programs. So far, a number of studies have reported the development of loop mediated isothermal amplification (LAMP) assays to detect *Wolbachia* in mosquitoes, but the methods still have some specificity and cost issues.

**Methodology/Principal findings:**

We describe here the development of a LAMP assay combined with the DNA strand displacement-based electrochemical sensor (BIOSENSOR) method to detect *w*AlbB *Wolbachia* in trans-infected *Ae*. *aegypti*. Our developed LAMP primers used a low-cost dye detecting system and 4 oligo nucleotide primers which can reduce the cost of analysis while the specificity is comparable to the previous methods. The detection capacity of our LAMP technique was 1.4 nM and the detection limit reduced to 2.2 fM when combined with the BIOSENSOR. Our study demonstrates that a BIOSENSOR can also be applied as a stand-alone method for detecting *Wolbachia*; and it showed high sensitivity when used with the crude DNA extracts of macerated mosquito samples without DNA purification.

**Conclusions/Significance:**

Our results suggest that both LAMP and BIOSENSOR, either used in combination or stand-alone, are robust and sensitive. The methods have good potential for routine detection of *Wolbachia* in mosquitoes during field surveillance and monitoring of *Wolbachia*-based release programs, especially in countries with limited resources.

## Author summary

Mosquito-borne diseases such as dengue, chikungunya, zika, and yellow fever are transmitted to humans mainly by the bites of *Aedes aegypti* mosquitoes. Controlling the vectors of these diseases relies mostly on the use of insecticides. However, the efficiency has been reduced through the development of insecticide resistance in mosquitoes. *Wolbachia* is an endosymbiotic bacterium that is naturally found in 40% of insects, including mosquitoes. The bacterium can protect its insect hosts from viral infections and can also cause sterility in insect host populations, therefore, providing an opportunity to use it for human disease control. Application of a *Wolbachia* trans-infected mosquitoes needs simple, rapid and sensitive methods for detecting the bacteria in released mosquitoes. In this paper, we develop the methods of LAMP and BIOSENSORS for detecting *w*AlbB *Wolbachia* in mosquitoes. Our positive LAMP reaction can be visualized by color change from violet to blue at a sensitivity of ≥ 10 pg of genomic DNA. When used in combination with the BIOSENSOR method, the sensitivity increases a millionfold without losing specificity. Our study suggests that both developed methods, either used in combination or stand-alone, are efficient and cost-effective, hence, they could be applied for routine surveys of *Wolbachia* in mosquito control programs that use *Wolbachia*-based approaches.

## Introduction

Dengue, chikungunya, zika, and yellow fever viruses, transmitted by the *Aedes aegypti* vector, continue to be a major health problem and affect human populations worldwide. Dengue is the most prevalent with an estimated 96 million symptomatic cases and 40,000 deaths every year [1]. Prevention of the transmission of these diseases, when vaccines have not yet been fully effective, depends primarily on two approaches, i.e., mosquito control and the interruption of human-vector contact such as using a mosquito net [2]. Historically, insecticides have been the primary means of mosquito control. However, the overuse and misuse of insecticides has resulted in several deleterious impacts on the environment and the emergence of insecticide-resistant mosquitoes [3]. Alternative vector control strategies are therefore important and need to be considered to effectively control the spread of these vector-borne diseases.

*Wolbachia* is an endosymbiont found intracellularly in about 40% of insect species [3]. The bacterium can manipulate host reproduction and inhibit virus intracellular replication [4,5]; hence it is potentially an effective alternative to traditional chemical pesticides. In mosquitoes, *Wolbachia* can induce cytoplasmic incompatibility (CI), a phenotype which results in the production of unviable offspring when uninfected females mate with *Wolbachia-*infected male mosquitoes. On the other hand, if *Wolbachia*-infected females mate with either infected or uninfected male mosquitoes, viable progenies harboring maternally transmitted *Wolbachia* will be produced. The effect of CI has received much attention, as it offers the potential application of *Wolbachia* in vector control. There have been a number of reports describing the stable establishment of *Wolbachia* in mosquitoes [6–8]. Since wild-type *Ae*. *aegypti* mosquitoes do not harbor *Wolbachia* [9], the introduction of *Wolbachia* into *Ae*. *aegypti* enabled disease control based on the CI and viral blockage properties. The use of *Wolbachia-*based approaches to reduce transmission of dengue, zika, and other viruses is currently being deployed and implemented widely in international programs in many countries [10,11]. Thus far many *Wolbachia* strains have been applied, including *w*Mel, *w*MelPop, and *w*AlbB. In Northern Australia, Florida, and Malaysia, *w*AlbB-infected *Ae*. *aegypti* has been successfully deployed, while *w*AlbA- and *w*AlbB-superinfected *Ae*. *aegypti* was successfully implemented together with the sterile insect technique (SIT) in the pilot population suppression trial in Thailand [12–15].

Although large-scale releases of *Wolbachia* trans-infected *Ae*. *aegypti* populations into the wild has been occurring in many countries, there remains critical issues with respect to the quality of the released mosquitoes. Surveillance of *Wolbachia* infection status is critical for the planning and deployment of proper mosquito control initiatives. Thus far, polymerase chain reaction (PCR) and qPCR techniques have been the gold standard methods used for detecting *Wolbachia* in mosquitoes [16,17]. However, the methods are laboratory based, require trained personnel, and use expensive instruments. Subsequently, loop-mediated isothermal amplification (LAMP), a highly sensitive and specific amplification of target DNA which requires only a heat-block, was developed and used for detecting *Wolbachia* in *Ae*. *aegypti*. To detect a diverse range of *Wolbachia* strains, LAMP primer sets were developed based on the 16S rRNA gene [18,19]. To evaluate the efficacy of the *Wolbachia* trans-infected mosquito interventions, LAMP primers specific to *w*AlbB and *w*Mel strains were developed based on the *Wolbachia* surface protein gene (*wsp*) [20,21]. For the *w*AlbB strain, a high-fidelity detection system using LAMP combined with oligonucleotide strand displacement (OSD) probes, and enhancement of the LAMP reaction speed using two loops together with a real-time fluorescence detection machine (Genie1), have been developed [20,22]. Based on DNA sequence analysis, the original primer set reported previously [20] was shown to be able to detect some variants of *Wolbachia w*Pip. Thus, the researchers suggested to label the primer loop with FAM to be the *w*AlbB vs *w*Pip OSD probe for differentiating *w*Pip [20]. Other researcher reported the application of this oligonucleotide sequence to be a loop WSP.FLP for increasing the detection speed [22]. Since applying many primers or using probe technology would increase the analysis cost, therefore using only 4 primers from other location(s) in the *w*AlbB *wsp* gene and applying a low-cost dye for LAMP analysis might be an alternative to have a more robust, sensitive, specific, but relatively low-cost LAMP detection method for the *w*AlbB-infected *Ae*. *aegypti* to detect *Wolbachia* in field released *Ae*. *aegypti* mosquitoes.

The LAMP products can be analyzed either by agarose gel electrophoresis or visual inspection of color or turbidity changes [23]. However, the disadvantage of the method is mis-diagnosis caused by a false positive or false negative. An alternative method to overcome the problem is the use of an electrochemical-DNA based biosensor, which employs gold-nanoparticles (AuNPs) to label nucleic acid [24–27]. AuNP-labeled reporter probes (RP) are hybridized with capture probes (CP) on magnetic bead nanoparticles (MBs, Fe_3_O_4_ nanoparticles). The target strand complementary to the CP displaces the AuNP-labeled RP and binds to the CP. A displacement reaction is accelerated by changing the temperature or salt concentration. The unbound AuNP-labeled RP is then separated from the MB-CP bound RP using magnetic separation. The amount of displaced AuNP-RP represents the amount of target DNA and can be detected by a differential pulse anodic stripping voltammetry (DPASV) technique performed using a potentiostat. Similar techniques have been applied to the diagnosis of the arboviral diseases, including the dengue, chikungunya, and zika viruses [28–31], but there has been very limited application in the mosquito sample [30]. The sensitivity of detection of these techniques were extremely high, with amounts as little as 1 PFU/ml being reported [30]. Some studies also showed the possibility of the technology being able to identify mosquito species and zika infection from mosquito salivary glands [29]. The extremely high sensitivity, together with the speed of detection allow the detection of low amount positive target as in the case of pooling samples in mosquito survey works. To our knowledge, no study has yet applied this technology to the detection of *Wolbachia* in mosquitoes.

In this paper, we describe the development of a combined LAMP and electrochemical-DNA based biosensor with strand displacement reaction method in order to detect *w*AlbB *Wolbachia* trans-infected *Ae*. *aegypti* mosquitoes. The conditions for LAMP and BIOSENSOR assays were also optimized as stand-alone techniques to be used for applications in other studies.

## Methods

### Ethics statement

The use of mosquito colony materials in this study was approved by the Faculty of Science, Mahidol University Animal Care and Use Committee (SCMU-ACUC) (Protocol No. MUSC64-005-554).

### Mosquito materials and genomic DNA extraction

Long-term laboratory rearing colonies including *Aedes aegypti* (Aae-JJ, from Jatujak, Bangkok, Thailand), *Aedes albopictus* (Aal-CH, from Chachoengsao, Thailand), *w*AlbB trans-infected Thai *Aedes aegypti* (*w*AlbB-TH, from Thailand), and *Culex quinquefasciatus* (Cq-BK, from Bangkok, Thailand) were used in this study. The *w*AlbB trans-infected Thai *Ae*. *aegypti* were generated using the direct microinjection technique as previously described [6,12]. Field collected mosquitoes used in the BIOSENSOR experiments were obtained in the evening by a human-landing catch (HLC) method, using a hand-held mouth aspirator, at a house in Suphanburi Province, Thailand. The containers filled with mosquito samples were kept in a freezer at -20°C until analysis.

Genomic DNA was extracted from approximately 200 mosquitoes (mostly females) using a crude boiling method [32]. Briefly, a single mosquito was ground in 100 μl of sodium chloride-Tris-EDTA buffer (STE; 10 mM Tris-HCl pH 8.0, 1 mM EDTA and 100 mM NaCl), heated for 10 min at 95°C, and centrifuged at 16000 rpm for 10 min. Supernatant was transferred to a new tube and used as a template sample in subsequent LAMP, PCR, and DNA sensor reactions. For the mosquito pooled experiment, 10 μl STE was used for each mosquito [20], and the DNA extraction was performed following the method described above. A pooled mixture was created by combining a single female *w*AlbB infected *Ae*. *aegypti* (*w*AlbB-TH) with specified numbers of uninfected female *Ae*. *aegypti* (24, 49, 74, or 99) to obtain the ratios of 1/25, 1/50, 1/75, and 1/100.

### LAMP primers and biosensor probe design

The sequence of the *wsp* gene of a *w*AlbB trans-infected Thai *Ae*. *aegypti* (NCBI accession number MZ325222) was applied for designing the LAMP primers. The sequence was identical to AF020059 *w*AlbB from *Aedes albopictus* (Houston strain) and MN307069 *Wolbachia* of *Aedes aegypti* isolate *w*AegB from the NCBI GenBank. This sequence was submitted to Primer Explorer v5 software (primerexplorer.jp/lampv5e/index.html, Eiken Chemical Co., Japan) to generate the potential primers used for the *w*AlbB LAMP detection. Several potential LAMP primer sets were generated. The primers were compared to various *wsp* sequences in the NCBI GenBank database. The DNA alignment was performed using MEGA 7.0.26 software with the default ClustalW algorithm [33]. The primer sets that bind to all *w*AlbB sequences but do not bind to many non-target *Wolbachia* strains were used for LAMP. The consensus region inside the LAMP priming site were used to construct biosensor probes (Table 1). A non-specific binding target was assessed by *in silico* analysis, where the primer sequences were compared with several *wsp* gene sequences from many strains in the NCBI database. All primers and probes were synthesized by Bio Basic Canada, Inc., Canada and Integrated DNA Technologies, USA, respectively.

**Table 1:** Oligonucleotide sequences of LAMP primers and target induced-DNA strand displacement probes used in this study.

Oligonucleotide	Sequence (5′–3′)	*wsp* Region
F3	CAAGAATTGACGGCATTGA	158–176
B3	ACCAATCCTGAAAATACTGC	355–374
FIP (F1c-F2)	CCATTTTATAACCAAATGCAGCACC AACCGAAGTTCATGATCCT	232–256, 189–207
BIP (B1c-B2)	GATGTTGAGGGACTTTACTCACAA ACACTGTTTGCAACAGTTG	271–294, 335–353
DP-WB-CP (Capture probe)	Biotin-TEG–TTATAACCAAATGCAGCACCACCAG	227–250
DP-WB-RP (Reporter probe)	Thiol Modifier C6 S-S–GGTGCTGCATTTGGTTATAA	232–246
dT_BP-5Bio (Biotin blocking probe)	Biotin-TEG–TTTTTTTTTT	
dT_BP-5SS (Thiol blocking probe)	Thiol Modifier C6 S-S–TTTTTTTTTT	

### LAMP assays

LAMP assays were performed in a total volume of 10–25 μl using *Bst* 2.0 WarmStart DNA Polymerase (New England Biolabs). The reagents, modified from Bhadra et al. (2018) [20], consisted of 1× Isothermal buffer (20 mM Tris-HCl, 10 mM (NH_4_)_2_SO_4_, 50 mM KCl, 2 mM MgSO_4_, 0.1% Tween 20, pH 8.8 at 25°C), 0.4 mM of dNTPs (10 mM each, Invitrogen, USA), 0.8 M Betaine solution (5 M, Sigma, USA), 2 mM MgSO_4_, 1.6 μM of each internal primer (FIP/BIP), 0.4 μM of each external primer (F3/B3), 6.4 units of *Bst* 2.0 DNA polymerase, 120 μM of hydroxy naphthol blue (HNB, Merck, Germany), and 4 μl DNA (diluted DNA supernanant 1:10 to obtain approximately 20–80 ng of DNA). HNB is a visualizing indicator of magnesium ion reduction due to the magnesium pyrophosphate formation by LAMP [23]. The developed LAMP assay was verified with *Ae*. *albopictus* naturally superinfected with *w*AlbA and *w*AlbB (Aal-CH), *Cx*. *quinquefasciatus* naturally infected with *w*Pip [34] (Cq-BK), wild-type *Ae*. *aegypti* mosquitoes which do not harbor *Wolbachia* [9] (Aae-JJ), and *w*AlbB trans-infected Thai *Ae*. *aegypti* (*w*AlbB-TH). The mixture was incubated at 65°C for 90 min, followed by 80°C for 10 min. The concentrations of DNA (20–100 ng), *Bst* 2.0 (1.6–8.0 U), and the reaction time (60–90 mins) varied depending upon experimental purposes as indicated in the Results Section. Any comparisons made to the previously reported LAMP analysis method [20,22] were in reference to the results of those specific studies.

### PCR reaction and analysis of gel electrophoresis

PCR detection of *Wolbachia* was performed according to the method previously described [35], using primers *wsp* 81F and 691R for general *Wolbachia* detection (~600 bp), primers 183F and 691R for *w*AlbB detection (~500 bp), and primers 328F and 691R for *w*AlbA detection (~380 bp) as a negative control. The reactions were performed using a final volume of 25 μl, and included 1.25 U *Taq* recombinant DNA polymerase (Invitrogen, USA), 1x PCR Buffer (w/o Mg), 3.75 mM MgCl_2_, 0.25 mM each dNTP (Invitrogen, USA), 0.5 μM each primer, and 1.0 μl template DNA. The amplifications were performed using a thermal cycler (T100 Thermo Cycler, Biorad, USA) with the following parameters: 1 cycle of 3 min at 94°C, 35 cycles of 45 sec at 94°C, 30 sec at 58°C, and 45 sec at 72°C, followed by 1 cycle of 10 min at 72°C. Five microliters of the PCR and LAMP products were mixed with 2 μl of loading dye, and were electrophoresed on a 2.0% (w/v) Agarose A gel (Biobasic, Canada) containing 0.2 μg/ml Ethidium Bromide (Sigma, USA) in 1xTBE buffer (pH 8.0) at 100 V for 40 min. DNA were visualized under UV light. The concentration of DNA was measured using NanoDrop One Microvolume UV-Vis Spectrophotometer (Thermo Fisher Scientific, USA).

### Functionalization of AuNPs conjugate with reporter probe

Preparation of AuNPs–reporter conjugate probe (AuRP) was performed using a salt aging method [27]. Briefly, 10 μl of 100 μM reporter probe DNA and 30 μl of 100 μM blocking probe (PolyT_10_) thiolated DNA were activated by using freshly prepared 10 mM Tris (2-carboxyethyl) phosphine (TCEP, Sigma-Aldric, USA). Then, the thiol-activated DNA was added into 1 ml of 40 nM AuNPs solution (DCN Diagnostics, USA) and incubated overnight at room temperature. After incubation, the solution of 10 μl of 500 mM Tris-acetate pH 8.2 and 100 μl of 1 M NaCl was added into the mixture and stored overnight at room temperature. The probes were obtained by centrifugation at 14,000 rpm for 30 min, followed by washing 3 times with 25 mM Tris-acetate pH 7.4, resuspended with hybridization buffer, and storage at 4°C until use.

### Immobilization of capture probe onto magnetic beads

The immobilization of the biotinylated capture probe (CP) on the magnetic bead (MB) (Dynabeads T1, Thermo Fisher Scientific, USA) was performed according to the manufacturer’s instructions. Briefly, a 100 μl volume of MB (10 μg/μl) was washed 3 times with 200 μl of 20 mM PBS pH 7.4, mixed with 4 μl of 100 μM capture probe, 12 μl of 100 μM blocking probe, and 184 μl of 20 mM PBS pH 7.4, and then incubated for 40 min at room temperature. The MB-bound probe was washed 3 times with 20 mM PBS pH 7.4, resuspended with 100 μl of 20 mM PBS pH 7.4, and stored at 4°C until use. This conjugation was subsequently called the magnetic bead conjugated capture probe DNA (MB-CP).

### DNA hybridization and DNA strand displacement reaction

The prehybridization step of MB-CP and AuRP was prepared as follows: 2 μl of MB-CP and 10 μl of AuRP were added into 18 μl of 20 mM PBS/0.1% SDS pH 7.4, and incubated for 20 min at 45°C in a water bath. The prehybridized MB-CP and AuRP was then washed 3 times with 20 mM PBS pH 7.4 using magnet collection. The pellet was used for a DNA strand displacement experiment. For DNA strand displacement, 30 μl of target DNA was added to resuspend the pellet and then incubate at 60°C for 30 min. A magnet was used to separate the target DNA bound to MB-CP from the unbound AuNPs-reporter conjugate probe. The five microliters of the supernatant was used for signal detection.

### Electrochemical detection of AuRP from DNA strand displacement reaction

Displaced AuRP was detected by using electrochemical measurement by the differential pulse anodic stripping voltammetry (DPASV) technique on a portable Palmsens 4 computer-controlled potentiostat with PSTrace version 5.7 software (Palmsens, The Netherlands). Two electrode systems, screened printed carbon electrodes or SPCE (Quesence, Thailand), consisting of two carbon tracks as a working electrode, reference electrode, and counter electrode in DPASV, were used. Five microliters of the desired sample was loaded onto a working electrode surface, followed by 50 μl of 1 M hydrobromic acid (HBr)/0.1 M bromine solution (Br_2_). For the pre-treatment step, the condition for deposition potential was -0.75 V and the deposition time was 100 sec. The step potential was set at 0.005 V, with a 0.1 sec interval time. The modulation amplitude was 0.1 V and the modulation time was 0.05 sec.

### Detection of nucleic acid derived from *w*AlbB

The products of the *w*AlbB LAMP reaction with a single female mosquito and PCR of *wsp* genes, *w*AlbA *wsp* gene, and *w*AlbB *wsp* gene in single female mosquito samples were applied to the biosensor detection. In addition, the unamplified macerated single female mosquito samples from the laboratory colonies (Aal-CH, Aae-JJ, *w*AlbB-TH) and those from the field collection were used in this study. The concentration of the DNA was determined by measuring the absorbance at 260 nm, using the NanoDrop One Microvolume UV-Vis Spectrophotometer (USA) in the DNA strand displacement platform, followed by differential pulse anodic stripping voltammetry (DPASV) detection. Five replications were performed for each detection.

## Results

### LAMP primer and probe design

The *wsp* genes of *Wolbachia* trans-infected Thai *Ae*. *aegypti* were sequenced. This sequence was compared to 17 *wsp* genes of *Wolbachia w*AlbB in mosquitoes from the NCBI database. The consensus region of 230 bp was submitted to PrimerExplorer software. The recommended LAMP primer sets were compared to 686 sequences of *wsp* genes from 66 *Wolbachia* strains [34–36] and all *wsp* genes of *Ae*. *aegypti* and *Ae*. *albopictus* in the database from Sanger or genome sequencing (data available upon request). The set of sequences that could bind to all *w*AlbB sequences and were different from most other strains were selected (Table 1).

All FIC, BIC, F3, and B3 primers with 6 priming sites have to bind altogether in order to produce massive DNA cauliflower complexes for LAMP detection [37]. By comparing the *wsp* gene sequences downloaded from NCBI database with the LAMP primers from our work and the *Wolbachia wsp* LAMP-OSD assay in the previous study [20], it was shown that our primers could bind to *Wolbachia* variants in mosquitoes in addition to *w*AlbB (non-target binding), including *w*AegB in *Ae*. *aegypti* (MN307069), *w*Pseu in *Aedes pseudalbopictus* (AF317487), *Wolbachia* in some *Armigeres subalbatu*s (KY457720) and *Armigeres obturbans* (KJ140130, KJ140132). The non-target bindings of LAMP primers found in this study were possible to be bound to the primer set in the previous study [20], except for *w*Pseu. The previous LAMP set could bind more to *w*Pip in *Cx*. *quinquefasciatus* (AF020061). However, this research also suggested to use their loop sequence for the OSD probe, which would eliminate the binding of *w*Pip. *In silico* analysis suggested that our newly designed LAMP primers would be highly specific to the *w*AlbB strain, with specificity comparable to the *w*AlbB LAMP primer set previously reported [20,22].

**Fig 1:**
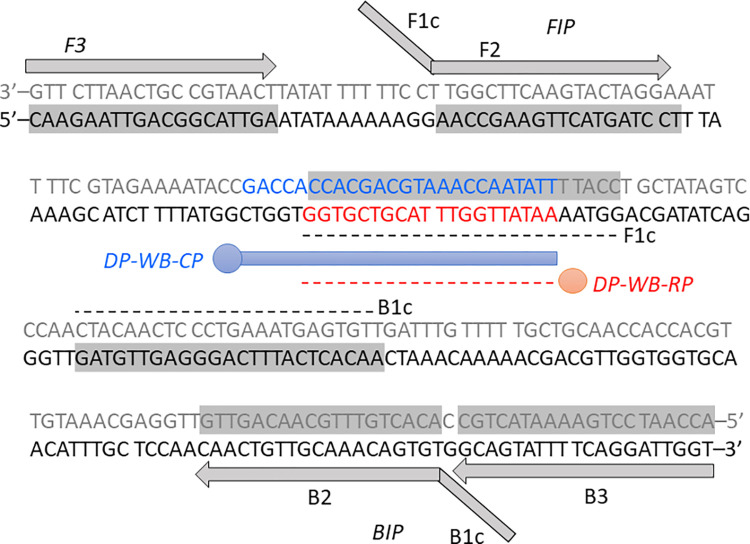
Schematic diagram demonstrates the LAMP primers and probe binding locations on the target sequence of the *w*AlbB *wsp* gene. Grey boxes indicate the primer sequences. Grey letters are the complementary sequence of the *w*AlbB sequence in the 5′-3′ direction. Blue and red fonts indicate the capture and reporter probes, respectively.

The new primers B3, FIP, and BIP in the present study had higher GC content (40–42%). The GC content was near the recommended range for good binding primers of 50–60% [38]. Our LAMP primers had a melting temperature in the range of 55.2–61.3°C, where the ΔG values of 3′ and 5′ ends were -6.24 to -4.07 kcal/mol and -5.69 to -4.02 kcal/mol, respectively, and the ΔG of dimer (minimum) formation was -2.16 kcal/mol. For the capture probe design, we selected the consensus region overlapping with the F1c binding area, so as to increase the attachment of the probe to the structures of complex LAMP products (Fig 1).

### Verification and optimization of LAMP assay

**Fig 2:**
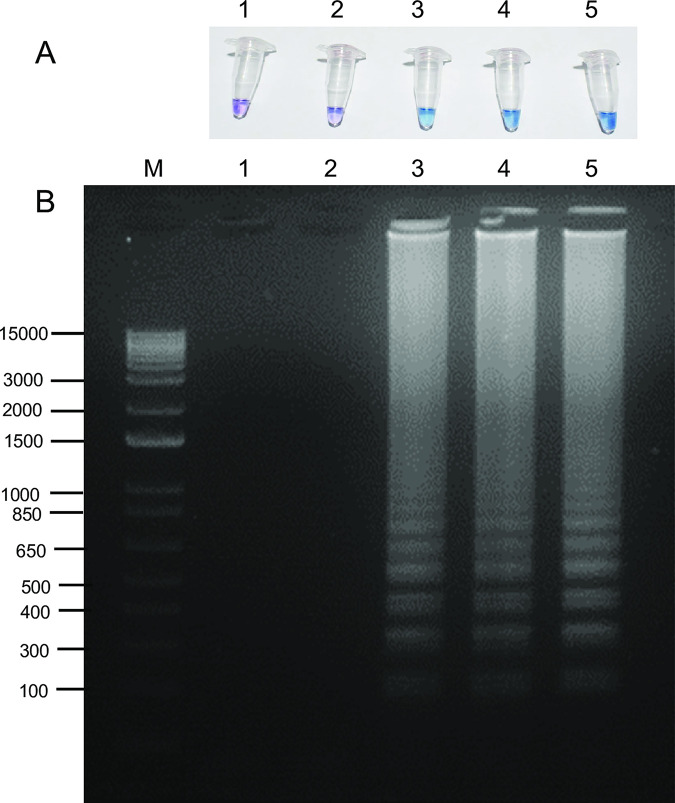
Detection of *Wolbachia w*AlbB gene in different mosquito species using LAMP assay with HNB indicator (A) and ethidium bromide-stained gel (B): (1) no template control, (2) wild-type *Aedes aegypti* (AegW, Aae-JJ), (3) *Wobachia* trans-infected Thai *Ae*. *aegypti* (AegB, *w*AlbB-TH), (4) *Aedes albopictus* (Alb, Aal-CH), and (5) *Culex quinquefasciatus* (Cq-BK). M is 1 kb plus DNA Ladder from Invitrogen. The condition for LAMP reaction was 6.4 units of *Bst* 2.0 DNA polymerase, 65°C for 60 min and 80°C for 10 min.

The developed LAMP primer set was used to examine the presence of *Wolbachia* in mosquito samples, i.e., *Ae*. *albopictus* (Aal-CH), *Cx*. *quinquefasciatus* (Cq-BK), wild-type *Ae*. *aegypti* (Aae-JJ), and *w*AlbB trans-infected Thai *Ae*. *aegypti* (*w*AlbB-TH) (Fig 2). The LAMP assay in Fig 2 clearly shows positive *Wolbachia* detection for the *Wobachia* trans-infected Thai *Ae*. *aegypti*, *Ae*. *albopictus*, and *Cx*. *quinquefasciatus*, as indicated by the blue color of HNB in the reactions and the presence of a ladder-like band pattern upon gel electrophoresis, while wild-type *Ae*. *aegypti* and the control reaction (no-template control (NTC)) were negative as indicated by a purple color in the HNB reactions and the lack of bands upon gel electrophoresis. These results agreed with the gold standard PCR method, suggesting the potential of these newly designed LAMP primers to detect *Wolbachia* infection in mosquitoes.

It is noteworthy that, as predicted from *in silico* analysis, all primers except for B3 could bind to the *wsp* sequence of the laboratory *Cx*. *quinquefasciatus* (MZ325223, Cq-BK). B3 contained one mis-matched base close to the 3′prime end. Although, theoretically LAMP will amplify only if the four primers are bound to a template with six priming regions, non-specific amplification may happen if the primers have the mismatch at the 3′prime end [39]. The detection of *Cx*. *quinquefasciatus* is an example of a non-target detection due to one mismatched base near the 3′prime end of the primer (Fig 2). However, *w*Pip was a variant of *Wolbachia pipientis* in different hosts to the *w*AlbB [35], therefore the detection of *w*Pip in *Cx*. *quinquefasciatus* by the *w*AlbB LAMP primers was possible. The previous LAMP-OSD assay also detected *w*Pip in *Cx*. *quinquefasciatus* in their study, although they created an additional probe to differentiate *w*Pip [20]. In addition, to verify the efficiency of indicating the presence of *w*AlbB strain, we repeated the test with 52 *Ae*. *albopictus* (Aal-CH), 30 *w*AlbB trans-infected Thai *Ae*. *aegypti* (*w*AlbB-TH), 31 wild-type *Ae*. *aegypti* (Aae-JJ), and 3 *Cx quinquefasciatus* (Cq-BK) (S1 Fig). All confirmed tests gave the results which were in good agreement with prior investigation, and that positive *w*AlbB LAMP assay was found in Aal-CH, *w*AlbB-TH, and Cq-BK mosquito samples, while the negative LAMP result was found in Aae-JJ.

To optimize LAMP detection, the concentration of the DNA template was examined. *Ae*. *albopictus* (Aal-CH) and *w*AlbB trans-infected Thai *Ae*. *aegypti* (*w*AlbB-TH) were used as positive controls. Wild type *Ae*. *aegypti* (Aae-JJ) and NTC were used as negative controls. The amounts of DNA template were varied between 20 and 100 ng (S2 Fig). The ladder-like bands were observed for *Ae*. *albopictus* and *w*AlbB trans-infected Thai *Ae*. *aegypti* samples for all template amounts, which were in contrast to the results of wild-type *Ae*. *aegypti* and NTC. Wild-type *Ae*. *aegypti* showed a darker blue-purple color closer to the positive control at a higher amount of DNA. However, at the DNA amount of 100 ng, the color from the wild-type *Ae*. *aegypti* reaction could not be differentiated from that of the positive reaction. Therefore, the amount of DNA should be controlled in a range of 20–80 ng, with the most recommended DNA amount being 20 ng. However, the quality of the DNA may be a matter of concern.

The LAMP assay was tested with different *Bst* polymerase concentrations from 1.6–8.0 units (S1 Table). The color development between the positive and negative control was more distinguished at higher concentrations of *Bst* (3.2–8.0 units). At 1.6 units of *Bst*, a false negative result was obtained for the *Wolbachia* trans-infected Thai *Ae*. *aegypti* (*w*AlbB-TH). Hence, *Bst* at concentrations of 3.2–6.4 units were recommended for cost saving and visual observation. However, in the present study, we used 6.4 units of *Bst*, as this amount of enzyme was used in many previous studies [18,20,40].

We also tried to vary the LAMP reaction time at 60 and 90 min for the 20 and 40 ng template DNA (S3 Fig). The HNB and gel results were positive for *Ae*. *albopictus* (P in S3 Fig, Aal-CH) and negative for NTC at both 60 and 90 mins for 20 and 40 ng DNA. However, the blue lavender color in the 60-min reaction of *Ae*. *albopictus* at 40 ng was ambiguous for visual observation. Therefore, 90 min was recommended. This is consistent with previous studies which also suggested 90 min for LAMP amplification [18,20]. Besides, the concentration of HNB used in this study was only 0.12 mM, which is 10-times less than that reported in the previous work [18]. However, the range of HNB dye concentration could be varied without affecting the LAMP reaction.

We also tested the stability of reagent mixture (- template) stored in a freezer (-20°C) to minimize errors caused by pipetting. Upon adding the DNA template, the LAMP reagent stored up to 30 days amplified the *w*AlbB trans-infected Thai *Ae*. *aegypti*, as indicated by the ladder-like band in an agarose gel (S4 Fig); although, the faint blue color could be observed when the reagent was stored up to 90 days. Based on our results, the reagent could be stored at -20°C for only 30 days.

**Fig 3:**
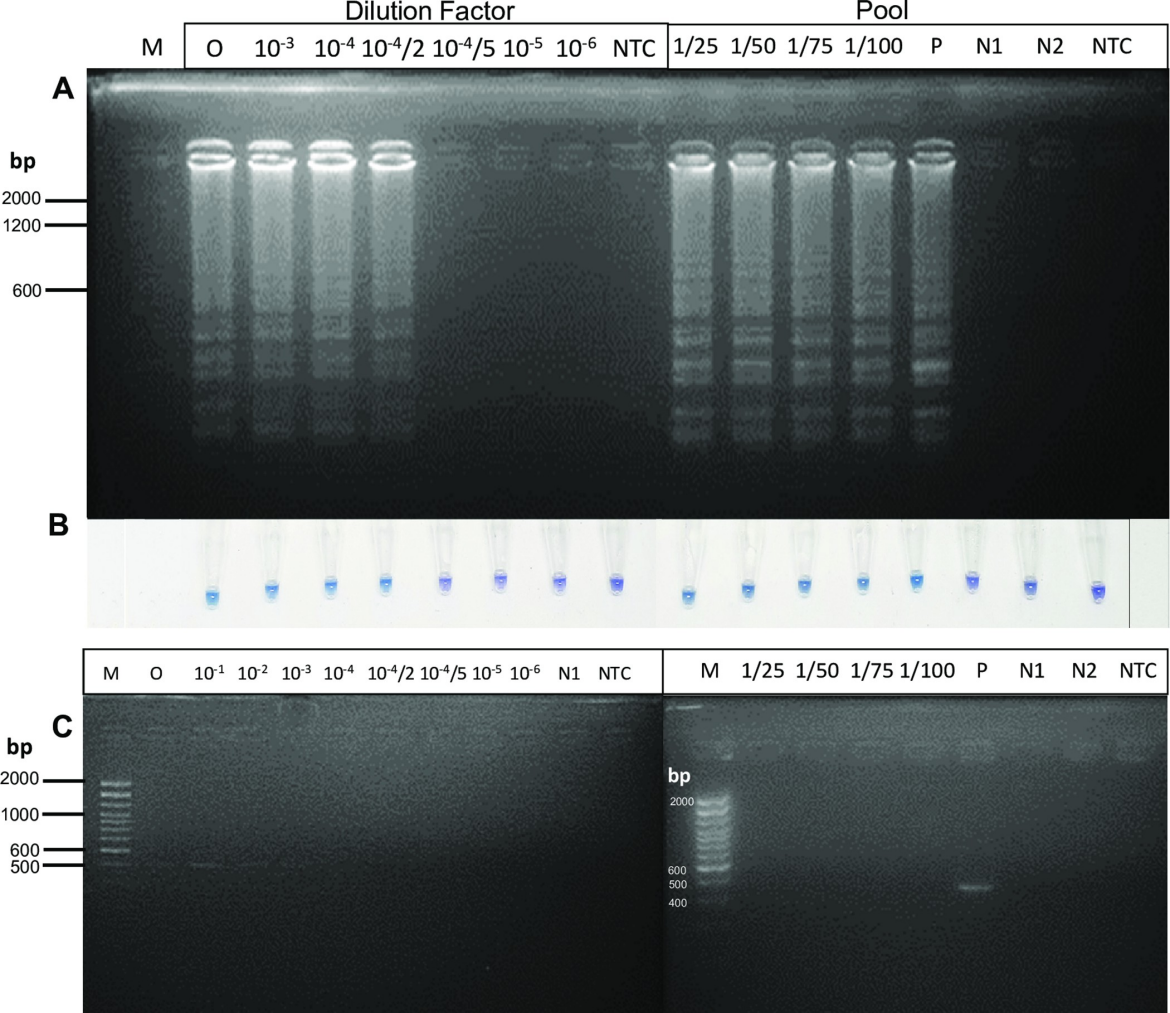
Sensitivity of LAMP (A and B) and PCR (C) detections with sample dilution experiment (Left) and mosquito pool sample experiment (Right). LAMP assay with ethidium bromide-stained gel (A) and HNB indicator (B) of a fold-dilution of an individual crude boiling *Aedes aegypti* (*w*AlbB-TH) sample (182 ng/μl, 260/280 = 1.90, 260/230 = 0.99) diluted 10^−1^–10^−6^ times (6.4 units of *Bst* 2.0 DNA polymerase, 65°C for 90 min and 80°C for 10 min, volume 10 μl). Polymerase chain reaction with *wsp* primers (691R and 183F) of the same dilution (C). (O) is a non-diluted original sample, (P) is *Wolbachia* trans-infected *Ae*. *aegypti* (*w*AlbB-TH), (N1) is wild-type *Ae*. *aegypti* (Aae-JJ), (N2) is 100 wild-type *Ae*. *aegypti* (Aae-JJ), (NTC) is no template control, (M) is Invitrogen 100 bp DNA Ladder. For mosquito pooled experiment, the ratio of 1/25 indicates the DNA extraction was performed with one *Wolbachia* trans-infected *Ae*. *aegypti* and 24 wild-type *Ae*. *aegypti*. Other pooled samples included 1/50, 1/75, and 1/100, which were equivalent to one *w*AlbB-TH mosquito in 49, 74, and 99 Aae-JJ, respectively.

The LAMP reaction was performed with diluted DNA from the *w*AlbB trans-infected Thai *Ae*. *aegypti* (*w*AlbB-TH, 182 ng/μl) in a total reaction volume of 10 μl with 1 μl DNA template. In Fig 3, LAMP could amplify the positive samples up to 20,000 folds (10^−4^/2 dilution), which was equivalent to 1.4 nM of DNA. Nevertheless, LAMP failed to detect DNA at a concentration below 0.56 nM. On another hand, PCR yielded a very faint band at a DNA concentration of 10^−3^-fold (or equivalent to 28 nM of DNA). LAMP had at least 20 times higher sensitivity than PCR. We concluded that the limit of detection (LOD) of LAMP in this study was 1.4 nM. In addition, the sensitivity of mosquito detection in pooled samples was tested. In Fig 3, LAMP could detect the presence of one *w*AlbB infected *Ae*. *aegypti* (*w*AlbB-TH) among 99 wild-type *Ae*. *aegypti* (Aae-JJ); while PCR could not detect the pool of one *w*AlbB-TH among 24 Aae-JJ. These results agreed with previous research [20,22].

### DNA sensors assay

**Fig 4:**
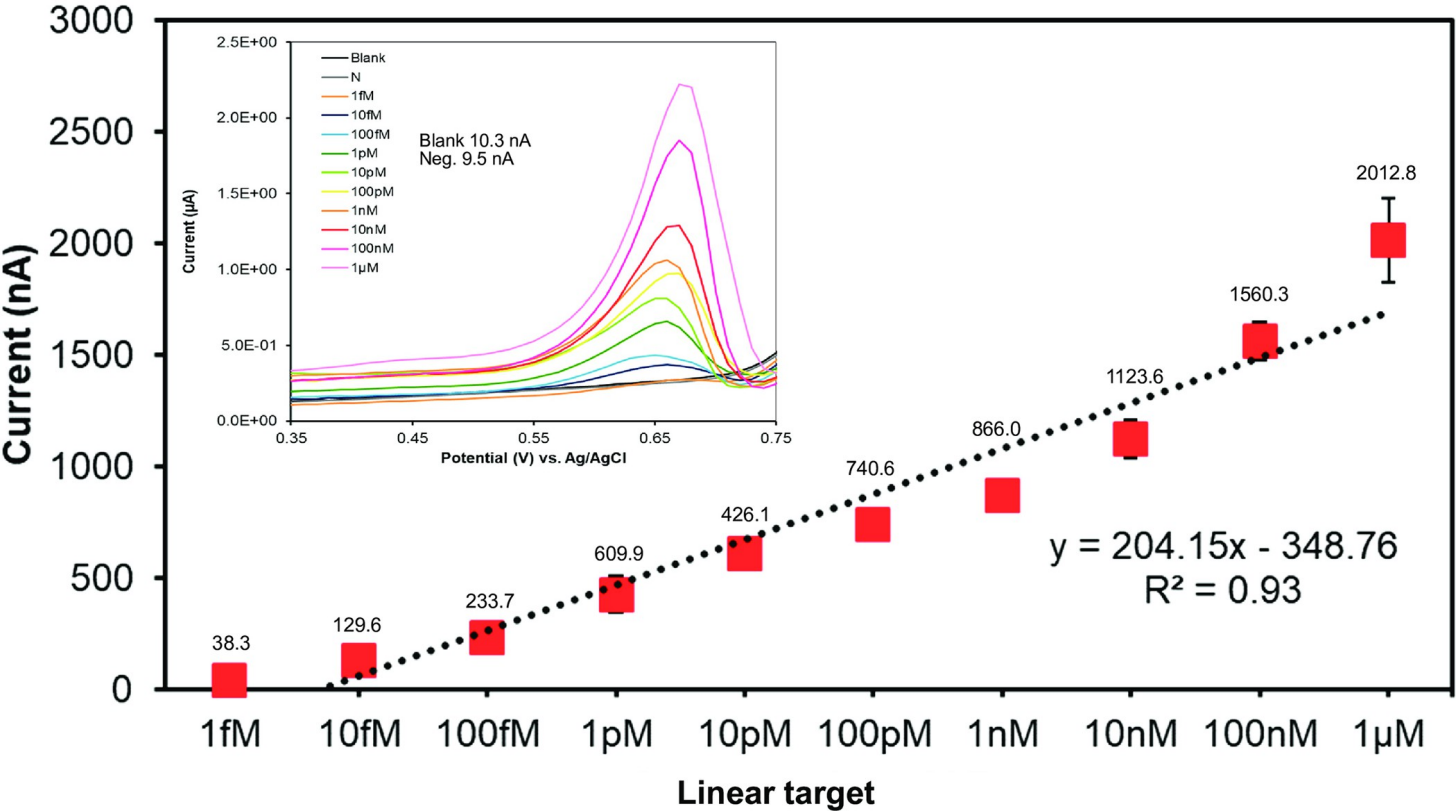
Calibration curve of synthetic *w*AlbB linear target strand displacement platform using electrochemical detection.

Biosensor was applied to increase the sensitivity of LAMP detection and to reduce ambiguity in LAMP visualization. Fig 4 shows the sensitivity of the strand displacement method with a synthetic *w*AlbB linear target using electrochemical detection. The results showed the LOD of 2.2 fM for the target DNA (5 Signals/Noise). The linear range was 1 fM to 1 μM (R^2^ = 0.93). The electrochemical target strand displacement platform had a much higher sensitivity than the LAMP and PCR techniques in a magnitude of 10^6^. Therefore, the sensitivity of *Wolbachia* DNA detection could be enhanced dramatically by using the electrochemical DNA sensors technology.

**Fig 5:**
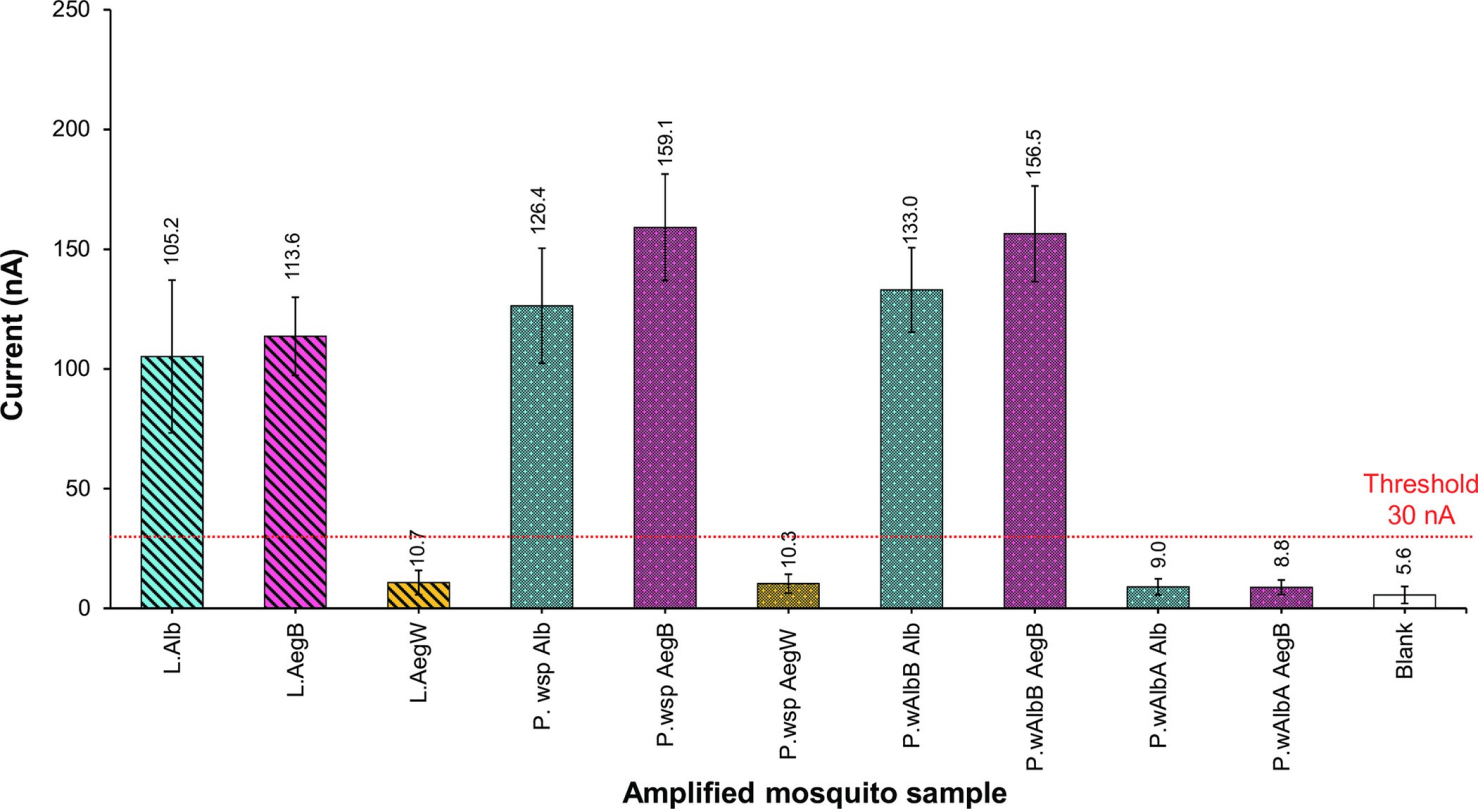
Electrochemical detection of *w*AlbB LAMP products (L) and PCR products (P). PCR reactions were performed for the general *wsp* gene (81F/691R), *w*AlbA gene (328F/691R), and *w*AlbB gene (183F/691R). Different mosquito species were included as follows: wild-type *Aedes aegypti* (AegW, Aae-JJ), *w*AlbB trans-infected Thai *Ae*. *aegypti* (AegB, *w*AlbB-TH), and *Aedes albopictus* (Alb, Aal-CH). Samples giving peak currents above 30 nA (approximately four-times blank signal) were considered positive. The bar charts represent the mean of 5 replications of DPV measurement and standard deviation was shown. Different patterns indicate either PCR or LAMP reaction products. Colors indicate mosquito colonies.

We applied this BIOSENSOR technology to the LAMP amplified samples (Fig 5-L). The results obtained from electrochemical detection clearly showed a good discrimination between *w*AlbB-infected mosquitoes (L.Alb and L.AegB) and non-infected mosquitoes (L.AegW). For the positive target, PCR products of *Wolbachia* detection with general *wsp* primers of the mosquitoes containing *w*AlbB (P.*wsp* Alb, P.*wsp* AegB, P.*w*AlbB Alb, and P.*w*AlbB AegB) were detected. The detection signal for negative samples from the mosquitoes without *w*AlbB (P.*wsp* AegW) were lower than the threshold value at 30 nA, approximately four-time blank signal, as shown in Fig 5. In addition, electrochemical detection with the *w*AlbB targeting capture probe did not respond to the PCR product of *w*AlbA (P.*w*AlbA Alb); though the mosquito DNA templates contained *w*AlbB. This test could effectively separate the detection of *w*AlbA and *w*AlbB.

**Fig 6:**
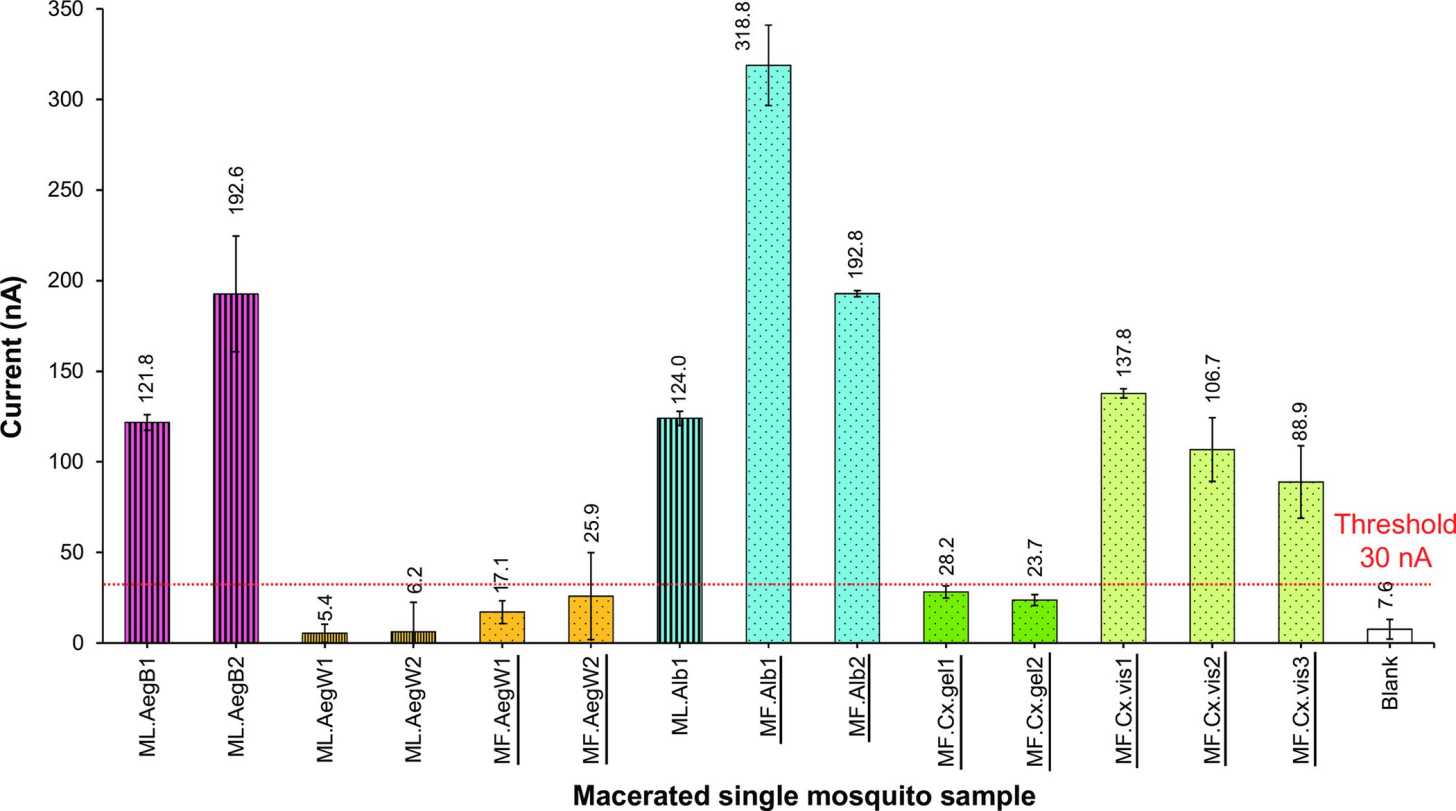
Electrochemical detection of macerated mosquitoes of laboratory colonies (ML) and field samples (MF). Different mosquito species were included as follows: wild-type *Aedes aegypti* (AegW, if ML: Aae-JJ), *w*AlbB trans-infected Thai *Ae*. *aegypti* (AegB, *w*AlbB-TH), *Aedes albopictus* (Alb, if ML: Aal-CH), *Culex gelidus (Cx*.*gel)* and *Culex vishnui (Cx*.*vis)*. Samples giving peak currents above 30 nA (approximately four-times blank signal) were considered positive. The bar charts represent the mean of 5 replications of DPV measurement and standard deviation was shown. Different patterns indicate either laboratory colony or field samples. Colors indicate different mosquito colonies or species.

Since we discovered high sensitivity of *Wolbachia* detection with the electrochemical DNA sensors, we applied it to test the presence of the *w*AlbB region directly in the macerated single mosquito samples without any *in vitro* amplification. Fig 6 demonstrates positive detection for *w*AlbB trans-infected Thai *Ae*. *aegypti* (ML.AegB1-2), *Ae*. *albopictus* (ML.Alb1 and MF.Alb1-2), and *Cx*. *vishnui* (MF.Cx.vis1-3), as well as negative detection for wild-type *Ae*. *aegypti* (ML.AegW1-2 and MF.AegW1-2) and *Cx*. *gelidus* (MF.Cx.gel1-2) from both laboratory colonies and field samples. As mentioned earlier, most of the studies reported no *Wolbachia* infection in wild-type *Ae*. *aegypti* and superinfection by *w*AlbA and *w*AlbB in *Ae*. *albopictus*. We obtained a negative result for the wild-type *Ae*. *aegypti* and a positive result for the *Ae*. *albopictus*. However, our biosensor platform detected a positive signal in *Cx*. v*ishnui* although the only available *wsp* gene sequence of *Cx*. *vishnui* (GQ469981) in the NCBI database did not match with the capture probe sequence. Previous studies reported that the infection of *w*Con belongs to the Supergroup B for *Cx*. v*ishnui* [9,41,42]. *Culex vishnui* may have different *wsp* strains or *w*AlbB that could bind to our probe. More *Wolbachia* survey in *Cx*. *vishnui* should be conducted in the future. The reported *wsp* gene sequences in *Cx*. *gelidus* (GQ469982.2 and AF317482) did not match with the sequence of the capture probe, and they belong to Supergroup A [41–42], which was consistent with the DNA sensor results obtained in this study. Although the detection might not be specific to only the *w*AlbB variant in *Ae aegypti*, other possible detected strains were in Supergroup B which are *Wolbachia pipientis* variants in different hosts [35]. In addition, a combination of the conventional morphological taxonomy and the molecular detection of mosquito species biomarkers [20,22] could simply differentiate the mosquitoes in *Aedes* and *Culex* groups with different *Wolbachia* strains.

## Discussion

This study developed a LAMP combined with the electrochemical detection of AuRP from a DNA strand displacement platform for detection of the *w*AlbB strain of *Wolbachia* bacteria. The methods, i.e., LAMP assay, LAMP plus BIOSENSOR assay, and BIOSENSOR assay alone, could be applied as surveillance and monitoring tools for *Wolbachia* trans-infected *Ae*. *aegypti* release programs. Since most studies thus far reported an absence of *Wolbachia* in the wild-type *Ae*. *aegypti* [9,17,41,43–44], it can be assumed that the *Wolbachia* in *Ae*. *aegypti* detected in the field surveillance and monitoring study is likely from the release programs.

Although there were few previous studies reporting the detection of natural infection of *Wolbachia* in *Ae*. *aegypti* [45–52], the *Wolbachia* detection methods in these studies employed only molecular approaches which are prone to contamination and may be subjected to horizontal gene transfer from the larvae or parasitic nematodes nearby or mis-identification of a naturally infected *Wolbachia* mosquito such as *Ae*. *albopictus*. Only two studies reported the successful establishment of laboratory colonies of *Wolbachia*-infected *Ae*. *aegypti* and demonstrated the inherited vertical transmission of *Wolbachia* to F2 [19] and F4 [45] generations. In another independent study [17], however, the cytoplasmic incompatibility and the molecular detection on the putatively *Wolbachia*-infected *Ae*. *aegypti* Las Cruces colony (New Mexico) of the previous work [19] were examined, but *Wolbachia* in this colony could not be found. Therefore, the authors concluded that the evidence of *Wolbachia* in *Ae*. *aegypti* was not compelling [17]. Regarding the intangible evidence of *Wolbachia* infection in natural *Ae*. *aegypti*, comprehensive monitoring of the infection status of *Wolbachia* should be continued, especially prior to the release of *Wolbachia* trans-infected mosquitoes. Our detection schemes using LAMP, BIOSENSOR, or a combination could serve this purpose well, as these methods are much more sensitive (Figs 3 and 4) than the conventional PCR method and can reduce the need for laboratory equipment and molecular biology specialists. However, LAMP and BIOSENSOR still need PCR approaches and precise morphological or molecular species detections for accurate confirmation of the *Wolbachia* detection in *Ae*. *aegypti* as shown in Fig 2 that *w*AlbB LAMP could amplified *w*Pip in *Cx*. *quinquefasciatus*.

Regarding the cost of analysis as shown in the S2 Table, LAMP reagents would cost around $0.6–$1.5/reaction, while PCR or qPCR costs around $0.7–$1.0/reaction. Approximate cost for a DNA sensor would be $2.0. The crude DNA extraction costs less than $0.5 per sample; though the DNA extraction kit might cost up to $10 per sample. In addition, LAMP required only a single heat block and perhaps a clean UV cabinet which cost around $1,200, while PCR will cost at least $5,500 and qPCR will be at least $40,000. The electrochemical detection of BIOSENSOR costs around $1,300. Notably, cost estimation varies greatly across geographic locations and inflation condition. The cost analysis here is mainly based on the prices in Thailand. Needless to say, both LAMP and BIOSENSOR, including the combination, would greatly increase the speed and sensitivity of *Wolbachia* detection compared to PCR based techniques. Therefore, these methods are very suitable for application in the field, where assessment of expensive molecular laboratory instruments is limited. However, LAMP cannot be used to quantify *Wolbachia* load, which is the prime ability of qPCR and biosensor. Each technique has its own advantages and disadvantages, and each will be more suited to certain scenarios. A comparison of different approaches for *w*AlbB *Wolbachia* detection in mosquitoes in terms of purposes, abilities, assay times, and costs were summarized in S2 Table.

A number of recent works, and also commercial products, support the possibility of preparing the LAMP reagent in freeze-dried form. Other studies showed that the lyophilized LAMP reagents remained stable for 24 months when stored at 4°C, 28 days at 25°C, and 2 days at 37°C, or at least 55 days at room temperature [53,54]. In addition, it is also possible to prepare the strand displacement biosensor reagent in the lyophilized prehybridization mixture. A previous study demonstrated that the prehybridization mixture stored at 4°C is stable up to 3 months without significant decrease in the current signal [27]. However, a decrease of 18% and 30% in the current signal was found in the mixture stored at 25°C and an outdoor ambient temperature (24–34°C) for 50 days, respectively. Further study is needed to apply the lyophilization technique to the *Wolbachia* detecting reagent so as to facilitate the studies which have limited resource settings.

The storage period of the dead mosquito bodies is also an important concern for a field survey. A previous work reported that 6 among 10 mosquito samples kept at -20°C for 7 days gave a positive color as compared to the color of the no-template-control [20]. For samples kept at 14 days and 21 days, 5 and 1 mosquito samples, respectively, among the 10 total samples each were positive. However, we could detect LAMP *w*AlbB positive in all trans-infected Thai *Ae*. *aegypti* (*w*AlbB-TH) mosquitoes (n = 6) stored at -20°C for 15 months (Fig 3); while in one batch of *w*AlbB-TH, LAMP reaction could amplify 2 among 3 mosquito samples kept at -20°C for up to 10 days (S5 Fig). However, the storage period of mosquito samples for LAMP analysis might be dependent on *Wolbachia* load in different culturing conditions and mosquito colonies. Detection of *Wolbachia* from dead mosquitoes stored in a dry condition up to 30 days at 26°C and 10 days at 37°C has been reported [22]. The use of the Genie1 III machine with real-time fluorescence detection was found to increase the sensitivity and reliability of typical LAMP detections with gel electrophoresis and color development [22]. The speed of detection could be increased to 6–12 mins using 6 LAMP primers (including 2 loops) [22]. For LAMP analysis, we found that DNA concentration is an important factor related to the accuracy of LAMP amplification (S2 Fig). Too high DNA content (>100 ng) resulted in a false positive detection by visual judgement. However, controlling the amount of DNA can be a challenging task in resource-limited settings. Without the ability to accurately quantify DNA, we recommend researchers to follow our DNA extraction protocol (1 mosquito in 100 μl of lysis buffer, diluted DNA supernanant 1:10) and LAMP reagent preparation procedure (4 μl in 25 μl reaction mixture). By following this ratio, the DNA concentration should be in the appropriate range of 20–80 ng DNA.

Since the tonality of HNB color (purple to blue) could give false positive or negative results by naked eyes observation, replications should often be conducted to minimize these false positive and negative results and to increase reliability of detection. This technique needs a comparison with the color of a negative control (mosquito without *Wolbachia w*AlbB) and an experienced observer for judgement. We also observed some ambiguous results which required confirmation by a gel electrophoresis technique. The electrochemical DNA sensor could extend the reliability of detection. However, if one applies only LAMP, other dye such as SYBR Green I (orange to green) [55], cresol red (red to yellow) [56], phenol red plus cresol red (purple to yellow) [57], or GeneFinder (red to green) [58] might be used. HNB has the advantage of cost (inexpensive), ease of use, and stability of color (2–3 weeks) [58,59]. LAMP is often prone to contamination; therefore, separation of a specific clean plastic cabinet for master mix preparation, and sample addition in a clean open bench-top, could reduce the contamination problem.

Based on the *In silico* analysis, one of our LAMP primer (B3) could not bind to the *w*Pip strain in *Cx*. *quinquefasciatus*, though we observed a positive result with the DNA sample from *Cx*. *quinquefasciatus* mosquito in Fig 2. There was one mis-matched base near the 3’ end of the B3 primer. Together with all other LAMP primers, it is likely to be due to non-specific amplification. The modification by adding a fluorescence probe, as in the case of the LAMP-OSD probe in a previous study [20], can increase the specificity because LAMP will visualize only the probe binding fragments. If the LAMP primer set in the previous studies [20,22] was applied with the low-cost dye such as HNB, our LAMP primer set should have a comparable specificity to the *w*AlbB as the sequence analysis of both primer sets showed only one mismatched to *w*Pip sequence near the 3’end. Since both *w*Pip and *w*AlbB are variation sequences of *Wolbachia pipientis* in different hosts (*Culex* and *Aedes*, respectively), which may have minor different characteristics [35], with the classical morphological or molecular species identification, it is possible to differentiate these variants. However, if it is important for some specific study, the modified *w*AlbB vs *w*Pip OSD in the other research work [20] is recommended, as the probe technology can increase the specificity. In fact, the previous LAMP-OSD primer modified *w*AlbB vs *w*Pip OSD [20], and the modified set with two loops addition were shown to be able to increase the detection speed [22], and were possible to be used together with low-cost dyes. Therefore, our LAMP primer would be an alternative method to other previous studies.

For future development of the biosensing method, a previous work reported the development of a lateral flow biosensor on the nitrocellulose membrane for visual detection of dengue virus using dextrin-capped AuNP as label [31]. A positive test generated a red test line on the strip, which enabled simple visual detection. Another interesting research work applied the aptamer–gold nanoparticle conjugates for the colorimetric detection of *Ae*. *aegypti* and zika virus from the mosquito salivary glands [29]. This might be further developed to be a non-invasive disease-vector monitoring tool such as wick-based feeding device and membrane-based sensor that may be visualized from the mosquito abdomen color or saliva absorbing membrane.

The LAMP primers and electrochemical biosensing method with strand displacement platform were successfully employed to detect mosquito samples containing the *w*AlbB strain of *Wolbachia* bacteria. The tests provided high sensitivity and specificity suitable for field surveys of mosquito distribution in *Wolbachia*-based projects using *w*AlbB trans-infected *Ae*. *aegypti* and for monitoring natural *Wolbachia* infections in wild-type *Ae*. *aegypti*. This knowledge has the potential to have a tremendous impact on the field of biological control of mosquito vectors, which in turn could reverse the increasing burden of arboviral illnesses worldwide.

## Supporting information

S1 Fig Detection of *Wolbachia w*AlbB gene using LAMP assays with Ethidium bromide-stained gel of different mosquito species including *Aedes albopictus* (Aal-CH) (1), *Aedes aegypti* (Aae-JJ) (2), and *Wolbachia* trans-infected Thai *Aedes aegypti* (*w*AlbB-TH) (3), and *Culex quinquefasciatus* (Cq-BK) (4). (N) is no-template control and (M) is Invitrogen 1 Kb Plus DNA Ladder. LAMP reaction was performed using 3.2 units of *Bst* 2.0 DNA polymerase at 65°C for 90 min.
(TIFF)

S2 Fig Detection of *Wolbachia w*AlbB gene using LAMP assays with Ethidium bromide-stained gel (A) and Hydroxy Naphthol Blue indicator (B) under different DNA template mass of the different mosquito species including *Aedes albopictus* (Aal-CH) (1), *Aedes aegypti* (Aae-JJ) (2), and *Wolbachia* trans-infected Thai *Ae. aegypti* (*w*AlbB-TH) (3). (N) is no-template control and (M) is Invitrogen 1 Kb Plus DNA Ladder. The sizes of DNA (bp) were indicated. LAMP reaction was performed using 3.2 units of Bst 2.0 DNA polymerase at 65°C for 90 min.
(TIFF)

S3 Fig Detection of *Wolbachia w*AlbB gene using LAMP assays with Hydroxy Naphthol Blue indicator (A) and Ethidium bromide-stained gel (B) under different incubation times for 60 and 90 mins with a DNA template of 20 and 40 ng. P is *Aedes albopictus* (Aal-CH) and N is no-template control. M is Invitrogen 1 Kb Plus DNA Ladder. The sizes of DNA (bp) were indicated. LAMP reaction was performed using 3.2 units of *Bst* 2.0 DNA polymerase at 65°C for 90 min.
(TIFF)

S4 Fig Detection of *Wolbachia w*AlbB gene using LAMP assays with Ethidium bromide-stained gel (A) and Hydroxy Naphthol Blue indicator (B) with a LAMP reagent mixture (- template) stored in a 20°C freezer. P is *Aedes albopictus* (Aal-CH) and N is wild-type *Aedes aegypti* (Aae-JJ), and NT is no-template control (NTC). Triplicate sets were performed but one example result was shown here. M is Invitrogen 1 Kb Plus DNA Ladder. The sizes of DNA (bp) were indicated. LAMP reaction was performed using 6.4 units of *Bst* 2.0 DNA polymerase at 65°C for 60 min and 80°C for 10 min.
(TIFF)

S5 Fig Detection of *Wolbachia w*AlbB gene using LAMP assays with Ethidium bromide stained gel (upper) and Hydroxy Naphthol Blue indicator (lower) with the dead mosquitoes stored at different temperatures including -20°C (A), 4°C (B), 27°C (C), and 37°C (D). 1–3 are *w*AlbB infected *Aedes aegypti* mosquitoes. P is *Aedes albopictus*. N is wild-type *Aedes aegypti*, and NT is no-template control (NTC). M is Invitrogen 100 bp or 1kb plus DNA Ladder. The sizes of DNA (bp) were indicated. LAMP reaction was performed using 6.4 units of Bst 2.0 DNA polymerase, 65°C for 60 min and 80°C for 10 min.
(TIFF)

S1 Table Detection of *Wolbachia w*AlbB gene using LAMP assays with Hydroxy Naphthol Blue indicator under different *Bst* 2.0 Polymerase concentrations of the different mosquito species. N is no-template control. LAMP reaction was performed at 65°C for 90 min.
(TIFF)

S2 Table Comparison of different approaches for *w*AlbB *Wolbachia* detection in mosquitoes. (TIFF)
