# Supplementary figures and images for: Simple, sensitive, and cost-effective detection of wAlbB Wolbachia in Aedes mosquitoes, using loop mediated isothermal amplification combined with the electrochemical biosensing method
Source: PLoS Negl Trop Dis. 2022 May 13;16(5):e0009600. doi: 10.1371/journal.pntd.0009600 (PMC9132313; doi:10.1371/journal.pntd.0009600)

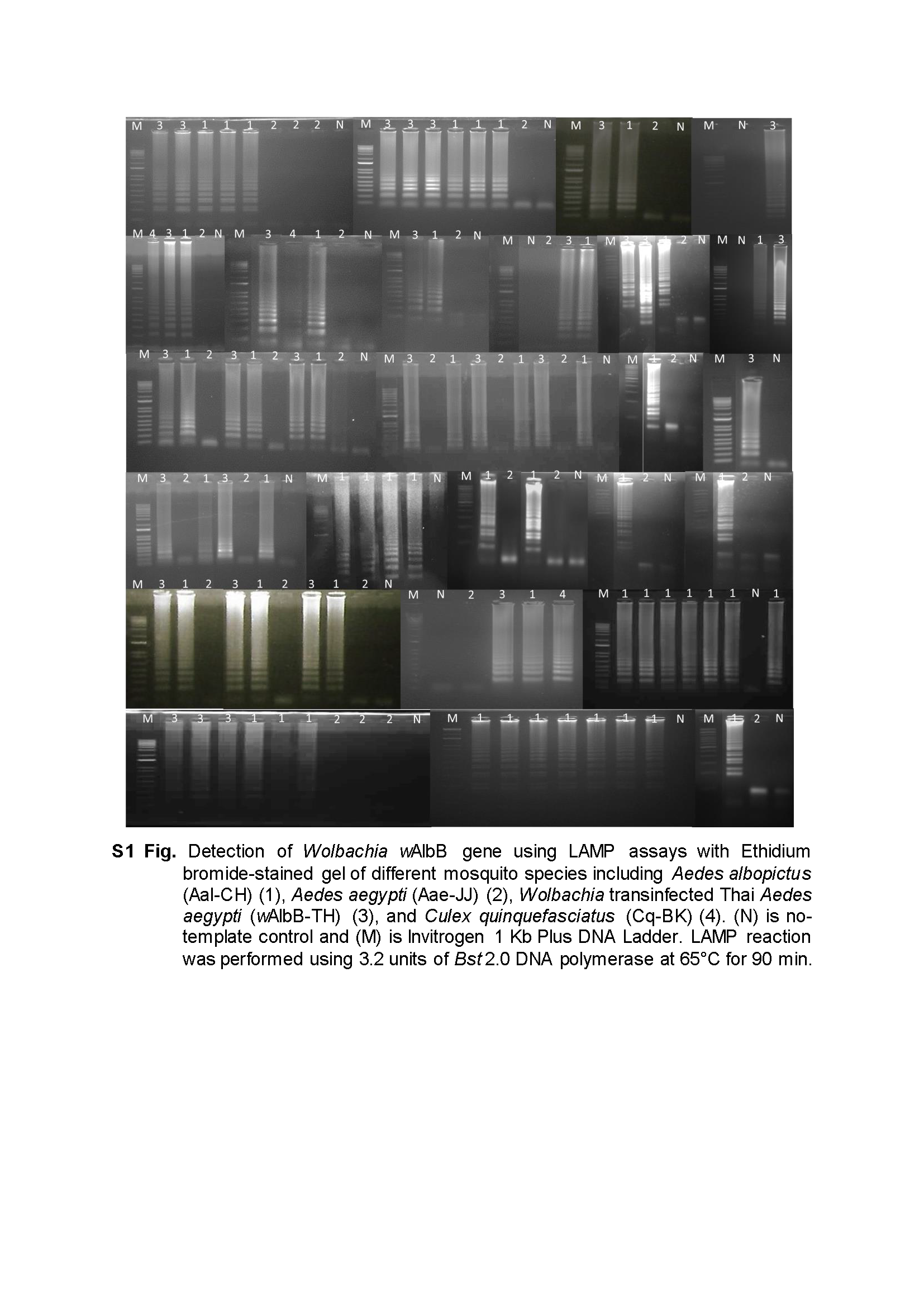

Supplement: S1 Fig — Detection of Wolbachia wAlbB gene using LAMP assays with Ethidium bromide-stained gel of different mosquito species including Aedes albopictus (Aal-CH) (1), Aedes aegypti (Aae-JJ) (2), and Wolbachia trans-infected Thai Aedes aegypti (wAlbB-TH) (3), and Culex quinquefasciatus (Cq-BK) (4). (N) is no-template control and (M) is Invitrogen 1 Kb Plus DNA Ladder. LAMP reaction was performed using 3.2 units of Bst 2.0 DNA polymerase at 65°C for 90 min. (TIFF) [file pntd.0009600.s001.tiff]

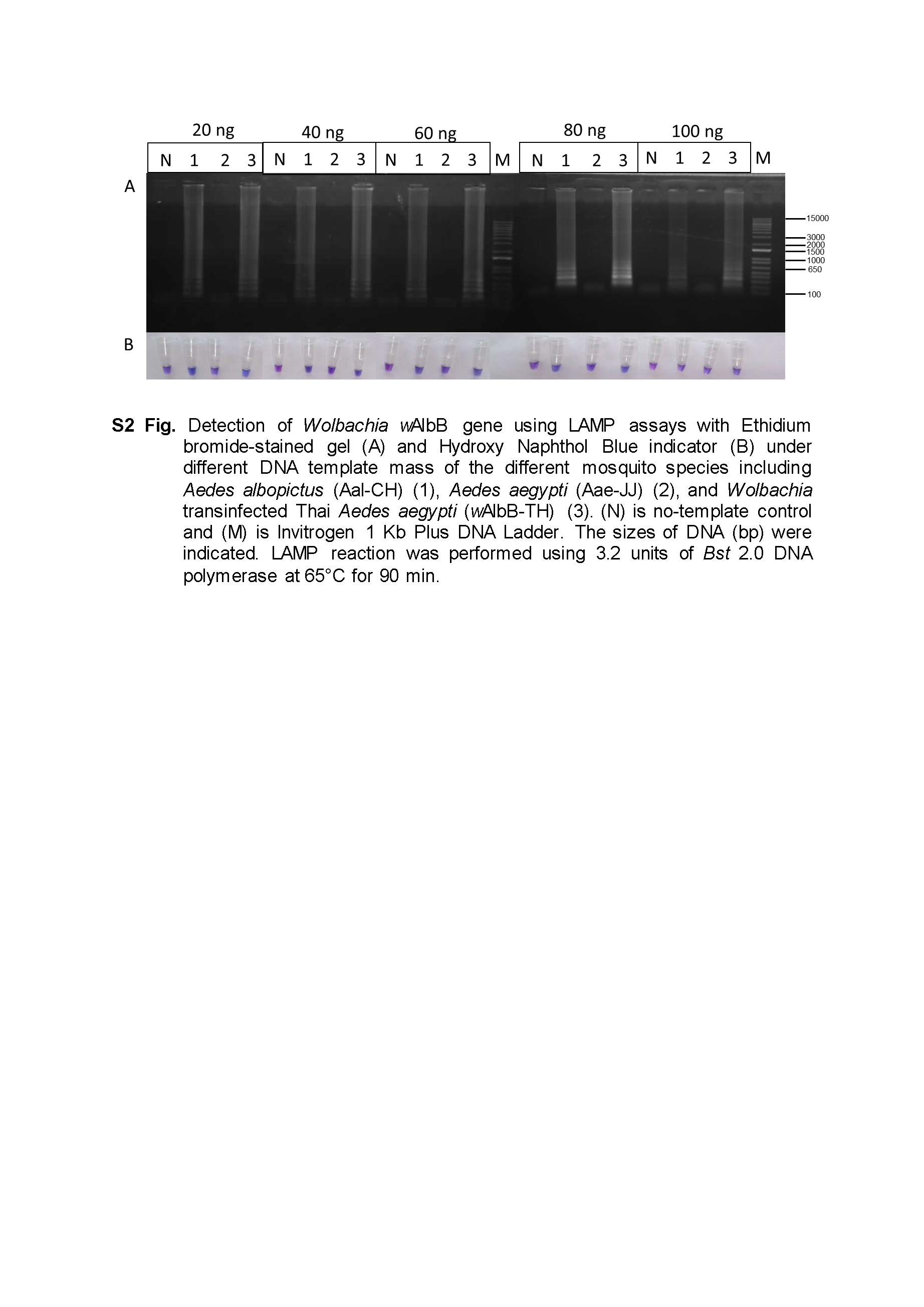

Supplement: S2 Fig — Detection of Wolbachia wAlbB gene using LAMP assays with Ethidium bromide-stained gel (A) and Hydroxy Naphthol Blue indicator (B) under different DNA template mass of the different mosquito species including Aedes albopictus (Aal-CH) (1), Aedes aegypti (Aae-JJ) (2), and Wolbachia trans-infected Thai Ae. aegypti (wAlbB-TH) (3). (N) is no-template control and (M) is Invitrogen 1 Kb Plus DNA Ladder. The sizes of DNA (bp) were indicated. LAMP reaction was performed using 3.2 units of Bst 2.0 DNA polymerase at 65°C for 90 min. (TIFF) [file pntd.0009600.s002.tiff]

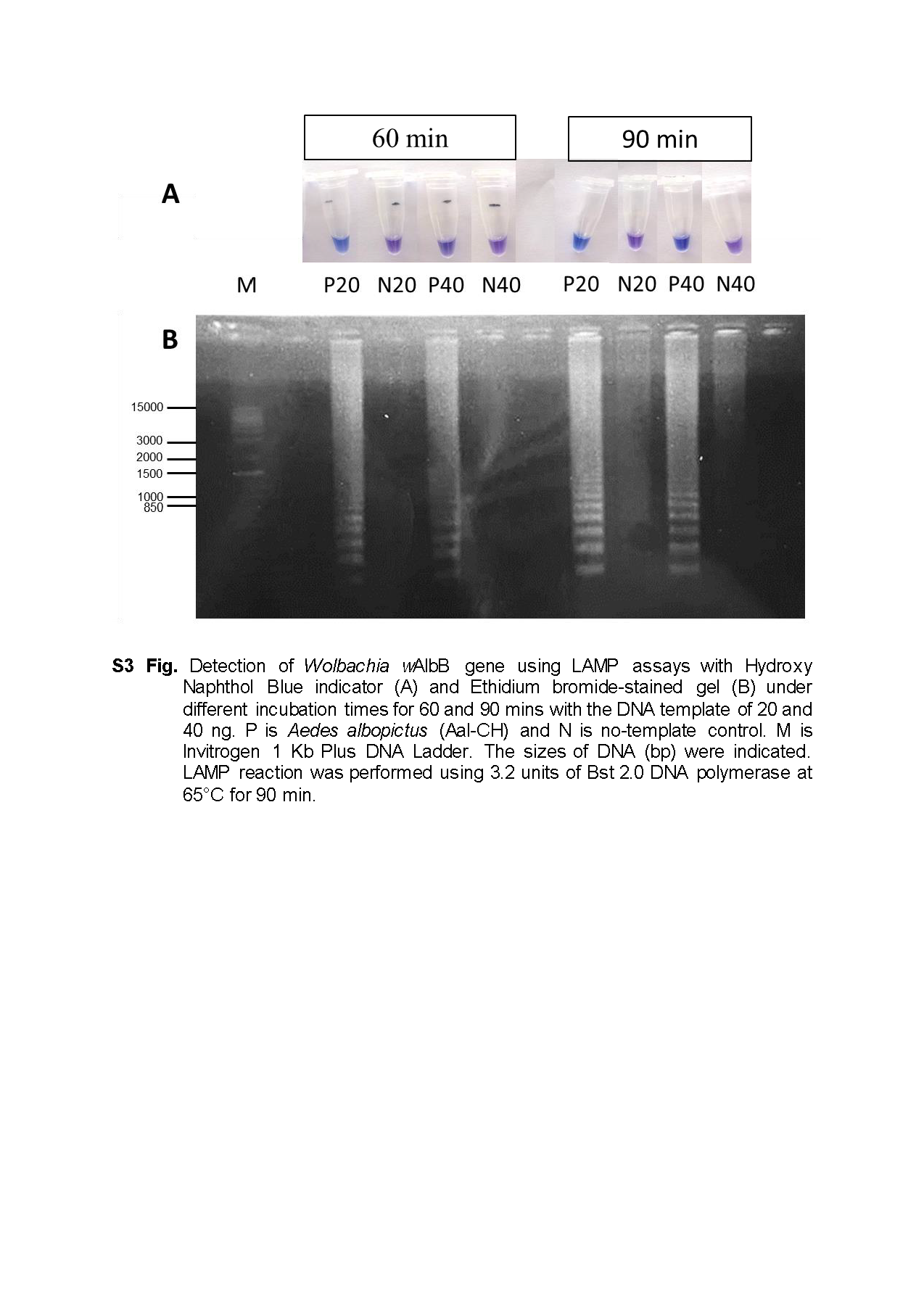

Supplement: S3 Fig — Detection of Wolbachia wAlbB gene using LAMP assays with Hydroxy Naphthol Blue indicator (A) and Ethidium bromide-stained gel (B) under different incubation times for 60 and 90 mins with a DNA template of 20 and 40 ng. P is Aedes albopictus (Aal-CH) and N is no-template control. M is Invitrogen 1 Kb Plus DNA Ladder. The sizes of DNA (bp) were indicated. LAMP reaction was performed using 3.2 units of Bst 2.0 DNA polymerase at 65°C for 90 min. (TIFF) [file pntd.0009600.s003.tiff]

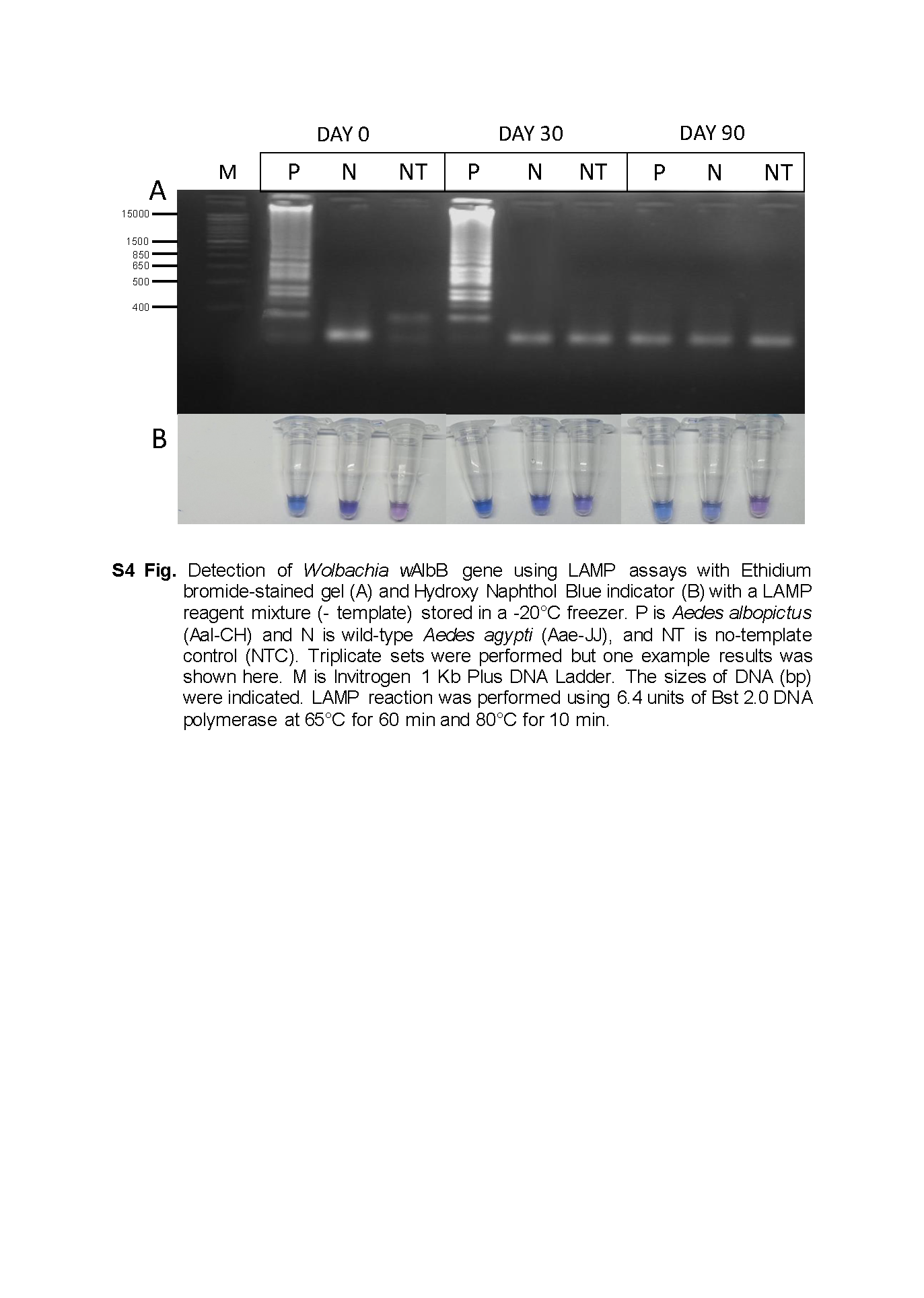

Supplement: S4 Fig — Detection of Wolbachia wAlbB gene using LAMP assays with Ethidium bromide-stained gel (A) and Hydroxy Naphthol Blue indicator (B) with a LAMP reagent mixture (- template) stored in a 20°C freezer. P is Aedes albopictus (Aal-CH) and N is wild-type Aedes aegypti (Aae-JJ), and NT is no-template control (NTC). Triplicate sets were performed but one example result was shown here. M is Invitrogen 1 Kb Plus DNA Ladder. The sizes of DNA (bp) were indicated. LAMP reaction was performed using 6.4 units of Bst 2.0 DNA polymerase at 65°C for 60 min and 80°C for 10 min. (TIFF) [file pntd.0009600.s004.tiff]

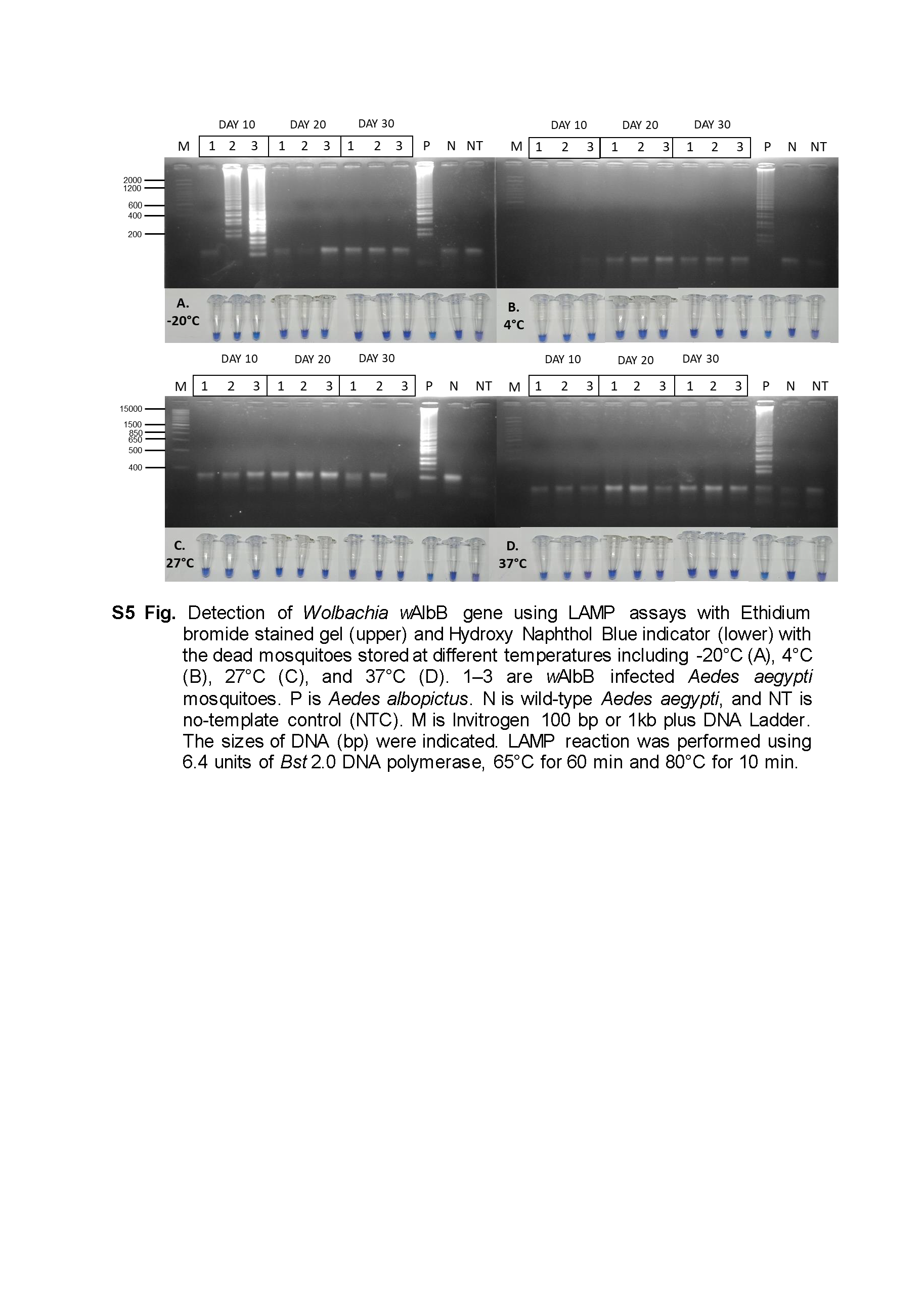

Supplement: S5 Fig — Detection of Wolbachia wAlbB gene using LAMP assays with Ethidium bromide stained gel (upper) and Hydroxy Naphthol Blue indicator (lower) with the dead mosquitoes stored at different temperatures including -20°C (A), 4°C (B), 27°C (C), and 37°C (D). 1–3 are wAlbB infected Aedes aegypti mosquitoes. P is Aedes albopictus. N is wild-type Aedes aegypti, and NT is no-template control (NTC). M is Invitrogen 100 bp or 1kb plus DNA Ladder. The sizes of DNA (bp) were indicated. LAMP reaction was performed using 6.4 units of Bst 2.0 DNA polymerase, 65°C for 60 min and 80°C for 10 min. (TIFF) [file pntd.0009600.s005.tiff]

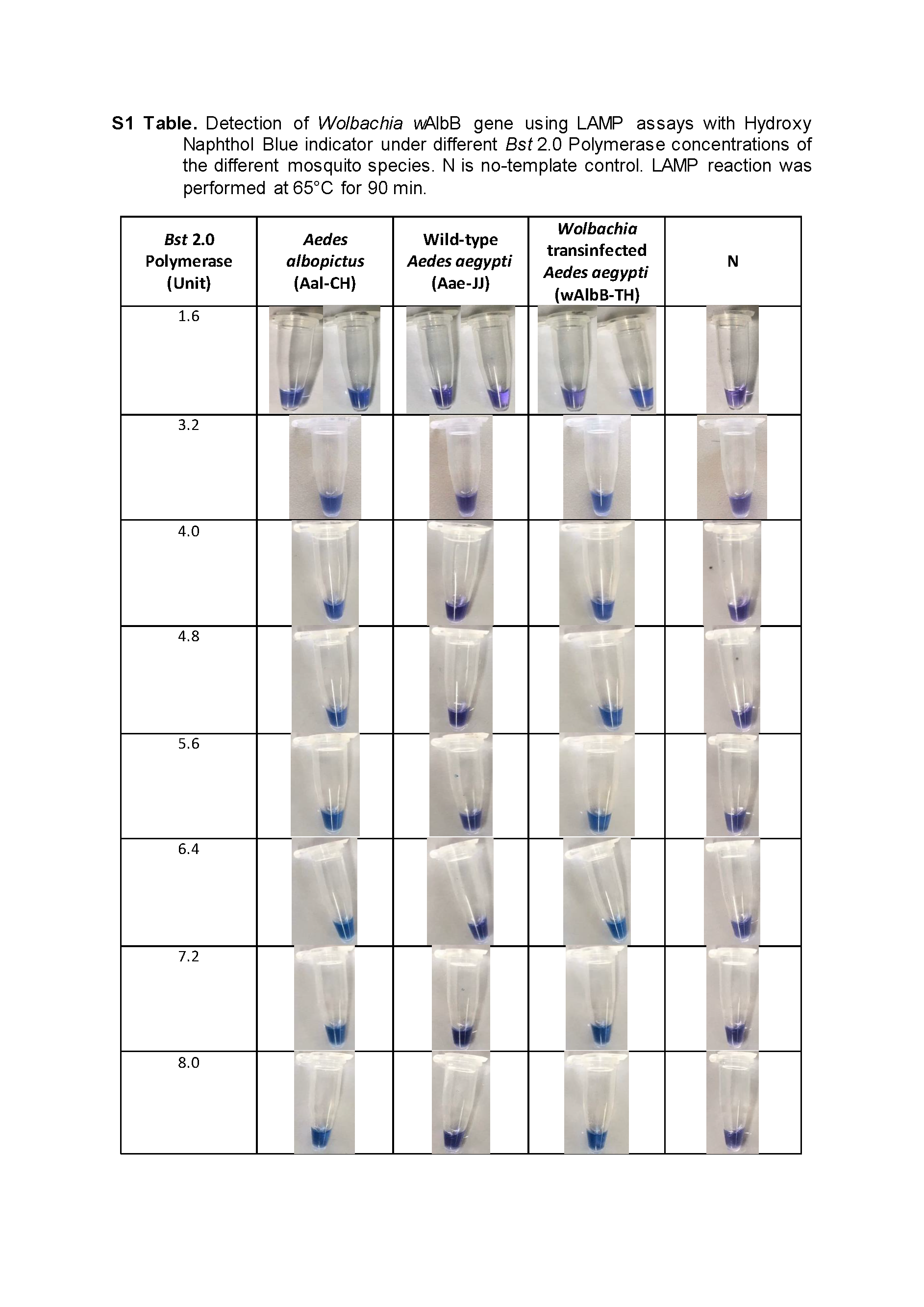

Supplement: S1 Table — N is no-template control. LAMP reaction was performed at 65°C for 90 min. (TIFF) [file pntd.0009600.s006.tiff]
